# Advancing Therapeutic and Vaccine Proteins: Switching from Recombinant to Ribosomal Delivery—A Humanitarian Cause

**DOI:** 10.3390/ijms252312797

**Published:** 2024-11-28

**Authors:** Sarfaraz K. Niazi, Matthias Magoola

**Affiliations:** 1 College of Pharmacy, University of Illinois, Chicago, IL 60612, USA; 2 DEI Biopharma, Kampala P.O. Box 35854, Uganda; dei@deigroupinternational.com

**Keywords:** in vivo mRNA, ex vivo mRNA, recombinant technology, therapeutic proteins, protein vaccines, antibodies, LNP, regulatory, intellectual property, ribosomes, bioreactors

## Abstract

Recombinant therapeutic and vaccine proteins have revolutionized healthcare, but there remain challenges, as many are awaiting development due to their slow development speed and high development cost. Cell-free in vivo ribosomes offer one choice, but they come with similar constraints. The validation of in vivo messenger RNA (mRNA) technology has been accomplished for COVID-19 vaccines. The bioreactors inside the body, the ribosomes, deliver these proteins at a small cost, since these are chemical products and do not require extensive analytical and regulatory exercises. In this study, we test and validate the final product. A smaller fraction of the recombinant protein cost is needed, removing both constraints. Although thousands of in vivo mRNA products are under development, their regulatory classification remains unresolved: do they qualify as chemical drugs, biological drug, or gene therapy items? These questions will soon be resolved. Additionally, how would the copies of approved in vivo mRNA protein products be brought in, and how would they be treated: as new drugs, generic drugs, or new biological drugs? Researchers are currently working to answer these questions. Regardless, these products’ cost of goods (COGs) remains much smaller than that of ex vivo mRNA or recombinant products. This is necessary to meet the needs of the approximately 6.5 billion people around the world who do not have access to biological drugs; these products will indeed serve the dire needs of humanity. Given the minor cost of establishing the manufacturing of these products, it will also prove financially attractive to investors.

## 1. Introduction

The discovery of proteins and understanding of their structures and functions in the human body emerged gradually over centuries, marked by transformative scientific breakthroughs. The term “protein” itself is derived from the Greek word “proteios”, meaning “primary” or “of first importance”, reflecting the early perception that these molecules were fundamental to life. Dutch chemist Gerardus Johannes Mulder is credited with coining the term in 1838 after analyzing organic molecules rich in nitrogen, including substances found in blood and egg whites [1]. This analysis pointed to a new class of compounds, distinct from other known organic materials, yet it would take more than a century to uncover the true complexity and significance of proteins.

The structure of proteins was first revealed through pioneering work in the 20th century. The linear sequence of amino acids within a protein, known as its primary structure, was first determined for insulin by Frederick Sanger in 1951 [2]. Sanger’s work was groundbreaking because it demonstrated that proteins are not just amorphous organic compounds but are composed of specific sequences of amino acids that dictate their function. For this discovery, Sanger was awarded the Nobel Prize in Chemistry, marking a turning point in protein chemistry. This initial success led to further structural discoveries. Linus Pauling and Robert Corey, in the early 1950s, elucidated key structural motifs like the alpha-helix and beta-sheet [3]. These secondary structures revealed that proteins fold into specific configurations driven by hydrogen bonds; a crucial insight into how proteins achieve their functional forms. In 1958, John Kendrew took the understanding of protein structure even further by using X-ray crystallography to solve the three-dimensional structure of myoglobin [4]. This was the first time a protein’s tertiary structure was visualized at an atomic level, showcasing the intricate folding patterns essential to biological function.

Simultaneously, the role of proteins in bodily functions began to unfold. By the mid-20th century, it was well understood that enzymes—specialized proteins—act as catalysts for metabolic reactions, facilitating nearly all chemical processes in the body [5]. The discovery that hormones, antibodies, and transport proteins also consist of proteins underscored their versatility, as these molecules were shown to be integral to signaling pathways, immune responses, and transport of essential molecules like oxygen. Through these advancements, proteins came to be viewed as central to nearly every cellular process, and their roles in health and disease became a focal point in medical research [6]. The advent of molecular biology, genomic sequencing, and proteomics in the late 20th and early 21st centuries further solidified proteins as the primary agents of biological function, offering a profound understanding of life at the molecular level and shaping modern medical and biological research.

These cumulative insights—from initial identification to structural elucidation and functional discovery—cemented proteins as essential biological molecules. Over the following centuries, research moved from rudimentary analysis to an in-depth understanding of proteins as dynamic, structured molecules responsible for the vast array of processes required to sustain life.

In addition to therapeutic proteins, protein vaccines constitute a crucial preventive healthcare management tool. Many of these products are awaiting development, but the high cost of recombinant manufacturing has withheld this progress.

The discovery that ribosomes synthesize proteins evolved through critical research efforts in the mid-20th century, particularly in the 1950s and 1960s. Early work on cell biology had identified ribosomes as small, dense structures within the cytoplasm, yet their function remained unclear. The concept of ribosomes as the site of protein synthesis began to crystallize in the 1950s, as researchers linked ribosomes with protein production in cells.

In 1955, cell biologist George Emil Palade observed ribosomes in the rough endoplasmic reticulum using electron microscopy. He noted that ribosomes were frequently associated with newly synthesized proteins, suggesting a possible role in protein assembly. Palade’s observations marked the first significant indication that ribosomes could be involved in protein synthesis. However, it was not until subsequent research, particularly in the early 1960s, that this role was definitively established [7].

The work of François Jacob and Jacques Monod in 1961 supported this by proposing the “messenger RNA hypothesis.” They hypothesized that messenger RNA (mRNA) carried genetic information from DNA to ribosomes, where proteins were synthesized according to this genetic code. Their model predicted that ribosomes and mRNA translate genetic information into functional proteins [8]. Following this, in the early 1960s, studies by Matthew Meselson and Franklin Stahl helped to clarify the mechanics of ribosomal protein synthesis, illustrating the critical role of mRNA as a template [9].

Finally, groundbreaking experiments by researchers like Marshall Nirenberg and Heinrich Matthaei, who cracked the genetic code in 1961, provided the first concrete experimental evidence that ribosomes use mRNA to assemble amino acids into proteins. By the mid-1960s, these discoveries firmly established ribosomes as the “machines” of protein synthesis, with their role in translating genetic information into proteins being widely accepted in the scientific community [10].

Palade was eventually awarded the Nobel Prize in Physiology or Medicine in 1974 for his contributions to understanding cellular structures and functions, including his discoveries on ribosome involvement in protein synthesis. Together, these efforts cemented the role of ribosomes as the sites of protein synthesis; a fundamental insight that revolutionized molecular biology and our understanding of cellular function.

These seminal studies collectively established the role of mRNA in protein synthesis. They provided a foundation for understanding the flow of genetic information from DNA to proteins; a core concept of molecular biology that opened two doors to therapies with proteins: recombinant DNA and synthetic mRNA, the topic of this paper.

However, another key aspect of introducing mRNA-based technology is the affordability of biological drugs that stand to transform the management of many life-threatening diseases, while their affordability and accessibility remain an issue. These drugs are more affordable in high-income countries (HICs), but their affordability tapers down as income is reduced. Low- and middle-income countries are home to over 6.5 billion people, and in these countries, the affordability of these drugs is anticipated to go down further by 1.5 to 2 times by 2030 [11].

## 2. Recombinant Proteins (RPs)

The human body produces thousands of proteins [12], which are responsible for cellular and extracellular functions vital to life. Initially, the only supply of several critical therapeutic proteins was through natural sources. For example, insulin was initially extracted from the pancreases of pigs and cows. This source was widely used for diabetes management, despite causing immune reactions in some patients due to its animal origin [13]. Human growth hormone (hGH) was sourced from cadaver pituitary glands for growth disorders. However, this posed a risk of transmitting prion diseases, such as Creutzfeldt–Jakob disease, due to its sourcing from human tissues [14].

Similarly, Factor VIII and Factor IX, crucial for treating hemophilia A and B, were derived from pooled human plasma, which presented a significant risk of contamination with blood-borne viruses like HIV and hepatitis C [15]. Albumin, a blood volume expander used in cases of hypovolemia and shock, was extracted from pooled human plasma, leading to similar contamination concerns and supply limitations [16]. Immunoglobulins, particularly intravenous immunoglobulin (IVIG), were obtained from large pools of human plasma and are still often sourced this way, as recombinant versions remain difficult and complex. Interferons, such as interferon-alpha and interferon-beta, were initially isolated from human leukocytes and fibroblasts for treating viral infections and multiple sclerosis, though in limited quantities due to sourcing challenges [17]. Additionally, Follicle-Stimulating Hormone (FSH), used for fertility treatments, was initially purified from the urine of postmenopausal women (urinary FSH) before recombinant FSH became available, which ensured a more stable supply [18]. The hepatitis B vaccine was another key therapeutic that was originally plasma-derived from infected individuals, posing a risk of contamination and supply shortages until recombinant versions became available in the early 1980s [19].

The history of recombinant technology dates back to 1970s [20]. This marked a significant milestone in biotechnology and the development of recombinant therapeutic proteins [21]. RPs join DNA from different species and subsequently insert the hybrid DNA into a host cell—often a bacterium or mammalian cell—to express the target protein; this molecular chimera was first created by researchers from UC San Francisco and Stanford in 1972 [22,23]. Stanley Cohen of Stanford and Herbert Boyer of UCSF received US patents in 1980. Boyer co-founded Genentech, Inc. in 1976. The Cohen–Boyer patents will eventually have more than 500 licensees to biotechnology and pharmaceutical companies and earn Stanford and UCSF more than USD 300 million in royalties [24]. The first recombinant drug approved by the FDA was human insulin, marketed as Humulin, in 1982. It was developed by Genentech and licensed to Eli Lilly. Humulin was the first therapeutic product created using recombinant DNA technology, where synthetic human insulin was produced in bacteria, revolutionizing diabetes treatment [25].

RPs are expressed in bacteria and Chinese hamster ovary cells [26,27,28]. RPs manufactured via modified DNA are expensive, restricting their accessibility across 80% of the world [29], despite their increasing numbers in the WHO Model List of Essential Medicines [30]. The regulatory constraints due to the safety and stability of RPs are also a significant issue [31] that restricts their wider availability.

As of 2024, over 100 US FDA-approved therapeutic proteins in 217 products have been approved. The global market for RPs is estimated to increase from USD 132.4 billion in 2023 to USD 203.6 billion by 2029 at a compound annual growth rate (CAGR) of 7.5% from 2024 to 2029 [32]. Their affordability is best viewed by the reimbursements made by CMS for Medicare patients, ensuring the lowest price paid for drugs. The cost per gram of Inotuzumab ozogamicin is over USD 25 million, and the same drug with a different antibody, is USD 2.7 million. The antibody with the lowest cost is about USD 350,000 per gram, while the manufacturing cost for these products is less than USD 100 per gram [26]. Table 1 lists the RPs approved by the FDA that can be manufactured using in vivo mRNA technology; however, it excludes chemically modified proteins such as antibody–drug conjugates, pegylated proteins, etc.

### Manufacturing Setup Cost of Recombinant Proteins

The cost of establishing a recombinant therapeutic protein manufacturing facility depends on factors such as production scale, geographic location, technology, and regulatory requirements. Initial capital investment can vary greatly, with small-scale facilities typically costing between USD 10 and 20 million, which is adequate for clinical trial production. In contrast, large-scale commercial facilities can cost USD 100 million to over USD 500 million [34,35]. Building or retrofitting a cleanroom facility that includes cell culture, purification, and formulation areas adds significant costs, from USD 5 million for smaller-scale facilities to USD 100 million for large commercial sites [35]. The equipment needed, including bioreactors, purification columns, and quality control tools, adds another USD 10–20 million for commercial-scale manufacturing [36,37].

Additional infrastructure investments, such as HVAC systems, Water for Injection (WFI), and energy-efficient systems, can range from USD 1 to 10 million [38]. Implementing automation and data management systems may also add USD 1–5 million to ensure streamlined processes and regulatory compliance. Once operational, annual costs for raw materials, utilities, consumables, and labor typically range from USD 10 to 50 million, with labor costs comprising 20–30% of total operating expenses [39]. Furthermore, adherence to cGMP standards and other regulatory requirements entails considerable investment in quality control and compliance, which adds several million annually [40].

Building and validating these facilities often requires 2–3 years, with indirect expenses like staff training and opportunity costs due to delayed production [41]. Single-use technologies and continuous manufacturing are recent innovations that reduce initial investment by eliminating some costly infrastructure and improving efficiency. However, they may increase operational costs in the long run due to consumable needs [39]. For a fully compliant, large-scale manufacturing facility, the cumulative investment, including setup and operational costs over 5–10 years, could exceed USD 500 million [42].

## 3. Ex Vivo mRNA Proteins (EMP)

Ex vivo ribosomal expression of proteins involves synthesizing proteins outside living cells using isolated ribosomes and essential translational components in a cell-free system. These systems replicate the intracellular environment by incorporating ribosomes, mRNA templates, tRNAs, amino acids, ATP, GTP, and other cofactors needed for protein synthesis. Ribosomes, typically extracted from organisms such as *E. coli*, wheat germ, or mammalian cells, are optimized for translation under controlled conditions. This approach provides several advantages, including rapid protein production, typically within hours, avoiding the time-intensive steps of cell culture. It is beneficial for synthesizing proteins that are toxic to cells or those requiring non-natural amino acids or isotopic labeling for structural studies [43,44].

Additionally, it enables precise control of reaction conditions, allowing for the study of ribosomal function and translation dynamics. Applications range from protein engineering, where unnatural amino acids can be incorporated, to structural biology, facilitating the production of isotopically labeled proteins for NMR spectroscopy or X-ray crystallography [45]. This technology is also pivotal in synthetic biology for constructing artificial ribosomes and in pharmaceutical development for rapid therapeutic protein screening. Despite their potential, ex vivo systems face limitations such as lower yields compared to vivo systems, higher costs due to expensive reagents, and challenges in producing large or complex proteins requiring extensive post-translational modifications [46]. Advances are ongoing, focusing on enhancing system stability, improving yields, and enabling the synthesis of more complex proteins, paving the way for on-demand protein production in applications such as personalized medicine and diagnostics [47]. EMP synthesis represents a versatile platform with immense scientific and therapeutic innovation potential.

However, the development costs for this process remain high due to validation and qualification, and the protein structure testing that adds the most cost to RPs remains intact in this process.

## 4. In Vivo mRNA Proteins (IMPs)

A much lower-cost solution to RPs is to deliver them using mRNA, essentially the innate bioreactors, the ribosomes, instead of ex vivo technologies to bring down the cost and time needed to develop protein drugs, as demonstrated by the faster development of the COVID-19 vaccines [48,49], now administered to billions of subjects [50,51].

The advantages of IMP technology for producing IMPS include the following [51]:

- It is faster and more efficient than RP technology and EMP. This means that development and production can happen more quickly. Complex and time-consuming cloning and expression in host cells are prerequisites for conventional recombinant technologies. On the other hand, IMP technology employs in vivo expression, removing all constraints of structural variability of recombinant proteins, which adds extensive cost to establish a reproducible process.
- mRNAs are smaller than plasmid DNA and never cross the nuclear membrane, staying in the cytoplasm for expression, eliminating genetic manipulation risks.
- They are more adaptable to producing complicated proteins, even ones that defy recombinant expression such as modification to make novel proteins, conjugates such as binding with transferrin protein, or expression of only minor parts of antibodies such as their scFv.
- In the future, the cell-based manufacturing systems used in mRNA and polymerase chain reaction (PCR) will be less susceptible to contamination from endotoxins and adventitious agents, reducing the safety risks.
- A short manufacturing cycle reduces costs significantly, and release testing is much simpler.
- IMP technology provides scalability and productivity benefits. One mRNA molecule can produce hundreds or even thousands of target protein molecules, making it highly efficient and reducing dosing, which brings better safety and lower costs.

These advantages of IMP technology are a promising and innovative approach to producing therapeutic proteins, with the potential for significant biopharmaceutical advancements [52].

### 4.1. Vaccines

RP proteins present one of the most significant segments of vaccines; Table 2 lists the FDA-approved recombinant vaccines.

These vaccines can be readily characterized by their structure, then a translational nucleoside sequence is generated to insert them into a standard mRNA product design [49,50,54]. While recombinantly produced vaccines will undergo extensive testing due to the possibility of the inherent structural differences among various batches, this not the case for mRNA-based delivery. Notably, the need for these vaccines is high, mainly in underdeveloped countries, where recombinant technology installations are almost impossible due to cost and expertise constraints. However, using modular, turnkey technologies for manufacturing [55], these vaccines can be produced at affordable CAPEX and OPEX worldwide. For the Least-Developed Countries (LDCs), it would not matter whether patents protect these vaccines. It is further noteworthy that there are many sources of funding available to LDCs to establish these technologies [56], which should encourage entrepreneurs in developing countries to take the lead.

### 4.2. Manufacturing Setup Costs of mRNA

**Figure 1:**
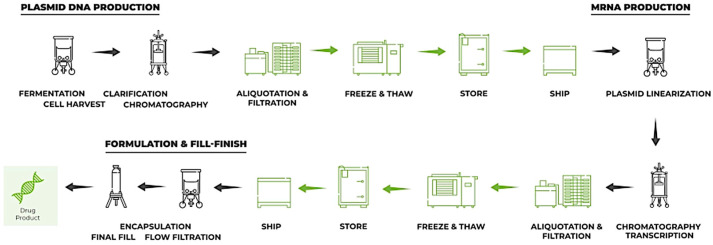
mRNA manufacturing setup technology.

mRNA technology has emerged as a promising approach to producing therapeutic proteins, offering several advantages over traditional recombinant technology or in vitro translation methods. Figure 1 shows the steps and details of the technology.

Establishing an mRNA manufacturing facility entails specific costs and considerations due to the unique nature of mRNA production processes and the technology required. Initial capital investment varies depending on the scale; small-scale facilities focused on research and clinical trials typically cost between USD 5 and 10 million, while large-scale commercial facilities—especially after the increased demand post-COVID-19—require investments from USD 70 to 200 million and can even exceed USD 250 million if they offer end-to-end production capabilities, including lipid nanoparticle (LNP) formulation and fill-finish lines [57,58]. Infrastructure and cleanroom construction or retrofitting, including specialized areas for plasmid production, in vitro transcription (IVT), purification, and LNP formulation, are essential components of these facilities, with costs ranging from USD 10 to 50 million [59]. Such cleanroom environments must meet regulatory standards, such as ISO 7 or 8, particularly for IMP synthesis and DNA preparation phases [60]. Furthermore, specialized equipment such as bioreactors for plasmid production, chromatography systems, and nanoparticle formulation devices typically costs around USD 15–25 million in a commercial setup, with additional expenses for quality control (QC) and quality assurance (QA) laboratories [52]. Given the delicate nature of mRNA, fill-finish systems—necessary to prevent RNA degradation—also require investment, adding USD 10–30 million to the initial setup for aseptic processes [61].

Annual operational costs, covering essential reagents, consumables, utilities, and labor, range between USD 5 and 25 million depending on production scale, with mRNA production being particularly reliant on high-purity reagents and enzymes, which contribute significantly to ongoing expenses [62]. Recruiting and training skilled staff with expertise in plasmid and mRNA synthesis, nanoparticle formulation, and quality assurance (QA) is a further cost driver, with labor typically representing around 20–30% of overall operational costs [63]. Quality control and regulatory compliance with standards from bodies like the FDA and EMA are also critical, requiring substantial, recurring expenditures for process validation, documentation, and batch testing [40]. Setting up and validating a complete mRNA manufacturing facility typically takes 12–24 months; some projects during the pandemic achieved this in under a year, but the standard timeline generally accounts for extensive validation processes [64]. Technological innovations, such as automated and single-use systems, have recently helped reduce upfront costs by replacing costly infrastructure with single-use bioreactors and modular production units. However, these technologies increase operational costs by relying on consumables [65]. End-to-end platforms for mRNA manufacturing, which integrate production with nanoparticle encapsulation, purification, and fill-finish processes, have further improved efficiency but demand significant initial investments. Setting up a fully functional, commercial-scale mRNA manufacturing facility, including all setup and operational costs over 5–10 years, is estimated to require an investment of USD 100–300 million (Table 3).

Table 4 lists the leading companies developing IMP products.

## 5. Intellectual Property

Despite the prevalence of mRNA technology patents, a specific infringement issue concerning the translation versus expression of the parent molecule in a cell remains unresolved. Whether mRNA-translated proteins can violate a patent is contingent upon several criteria, including the patent’s scope and particulars, jurisdiction, and the specific use of the mRNA-translated protein. Patents may encompass mRNA sequences, the methodology for protein translation utilizing mRNA, and the resultant proteins. Patent holders may, in certain instances, issue licenses for applications. Certain jurisdictions may implement research and experimental use exemptions, albeit interpretations differ. Geographical variations in patent legislation can influence the assessment of infringement.

One specific area is the lipid nanoparticle (LNP) formulations that many patents cover. A simple solution is to use a formulation that the FDA approved, which has an expired patent. An older FDA-approved RNA product that utilizes lipid nanoparticle (LNP) delivery is *Onpattro* (patisiran), developed by Alnylam Pharmaceuticals. Approved in 2018, it is a small interfering RNA (siRNA) therapeutic for treating hereditary transthyretin amyloidosis (hATTR). The LNP system used in Onpattro initially relied on technology patented by Arbutus Biopharma, explicitly involving the MC3 lipid, a key component for delivering RNA to cells. Alnylam’s use of MC3 was licensed from Arbutus. Although the MC3 patent was crucial for Onpattro, it will expire soon, making it one of the first LNP formulations potentially moving toward an unpatented status [66].

This expiration is significant because it could open opportunities for other companies to use similar LNP technology for RNA-based therapies without requiring a license from Arbutus. However, due to the rapid advancements in RNA delivery technologies, many companies, including Alnylam, Moderna, and BioNTech, have since developed their own proprietary LNP formulations to navigate patent constraints and address the limitations of earlier LNP systems used in siRNA and mRNA applications [67].

Onpattro’s regulatory journey also helped to establish foundational guidelines for RNA-LNP product approvals, influencing how components are classified as either active substances or novel excipients, depending on their specific biopharmaceutical effects and regulatory region. This categorization of LNP components remains essential in FDA filings. It can vary, affecting the patent landscape across different jurisdictions and products as newer RNA therapies emerge and older LNP patents expire [68].

It is noteworthy that the newer technologies claim higher efficiency. Still, when mRNA is used to express therapeutic proteins, efficiency is not as crucial, since each mRNA molecule can produce thousands of protein molecules.

Alnylam’s siRNA-based therapeutics beyond Onpattro involve several small interfering RNA (siRNA) products, like Givlaari and Oxlumo, utilizing proprietary LNPs developed after MC3. Although these formulations are still under patent, the advancements were influenced by expired and expiring patents from early lipid systems in non-mRNA drugs.

Alnylam’s use of LNPs in small interfering siRNA treatments for diseases like hypercholesterolemia also relied on foundational patents from Arbutus, although these earlier lipid structures have continued to evolve. Some of the earliest lipid-based drug delivery patents that expired have influenced regulatory precedents and facilitated newer generations of RNA products, allowing for innovation without re-licensing those expired technologies.

An exciting opportunity is available for UN-classified Least-Developed Countries (LDCs), where pharmaceutical patents do not apply until 2033 [69]. These 46 countries are Afghanistan, Angola, Bangladesh, Benin, Bhutan, Burkina Faso, Burundi, Cambodia, Central African Republic, Chad, Comoros, Democratic Republic of the Congo, Djibouti, Eritrea, Ethiopia, Gambia, Guinea, Guinea-Bissau, Haiti, Kiribati, Lao People’s Democratic Republic, Lesotho, Liberia, Madagascar, Malawi, Mali, Mauritania, Mozambique, Myanmar, Nepal, Niger, Rwanda, São Tomé and Príncipe, Senegal, Sierra Leone, Solomon Islands, Somalia, South Sudan, Sudan, Tanzania, Timor-Leste, Togo, Tuvalu, Uganda, Vanuatu, Yemen, and Zambia [70]. Given the realization that reproducing a chemical product such as mRNA is relatively safe, it is an opportunity for these countries to grow mRNA technology for their domestic needs. Technology deployment has become easier with the availability of ready-to-operate portable facilities that further reduce the time and cost of establishing mRNA products easier and faster. Notably, many mRNA vaccines will arrive soon, and the 46 LDCs should be able to supply an identical product without waiting or paying excessive amounts [71]. For example, the COVID-19 vaccine was selling for approximately USD 30 per dose, while its manufacturing cost was less than USD 0.20 [49]. The ease of making mRNA vaccines just as effective as those approved in Western countries makes it a lucrative opportunity for LDCs.

## 6. Talent Access

While the developing world will be the greatest beneficiary of the RibPs technology, A skilled workforce will still be needed to manufacture these products. However, the difference between what is required to adopt recombinant technology and ribosomal technology is remarkably less. The recombinant technology involves equipment such as the upstream bioreactors that require engineering and manufacturing skills to manufacture a biological drug with consistent quality; testing it is another highly complex process requiring such high-skill equipment such as mass spectroscopy, NMR and others, while the mRNA product is a chemical that is easily analyzed by routine chromatography and there are no upstream conditions that need to be manipulated since the production of pDNA is a non-GMP process; it is intended to produce DNA that is then split into RNA. With the advancements coming soon, this step will also be removed and replaced with PCR. The most critical aspect of ensuring that the product meets the release specification is that it is much easier and that the workforce is likely to be available or trained compared to the recombinant methods.

## 7. Regulatory

Regulatory scenarios are now arising, such as the conflict between the nature of mRNA and a chemical drug. Still, mRNA technology is listed in the FDA’s gene therapy classification. Would a copy of an approved mRNA product with an identical sequence be considered generic? This remains to be resolved.

As mRNA technology gains prominence, regulatory bodies formulate rules and frameworks for assessing and approving mRNA-based medicines, yet this remains ambiguous. These regulatory systems must ensure safety and monitor harmful effects [72]. The International Council for Harmonisation of Technical Requirements for Pharmaceuticals for Human Use (ICH) Q3A and B recommendations address “impurities in new drug substances and products generated through chemical synthesis”. It is important to emphasize that these rules do not apply to chemically produced oligonucleotides, such as ASOs and siRNAs. Furthermore, it is worth noting that they do not adhere to the ICH Q6A guideline [73] on “specifications: test procedures and acceptance criteria for new drug substances and new drug products: chemical substances”. The European Medicines Agency (EMA) categorizes mRNA products as gene therapy medical products (GTMP) or vaccines according to their functionality.

On the other hand, the EMA’s classification scheme classifies antisense oligonucleotides and RNA interference (RNAi) as chemicals [74]. “Gene therapy”, precisely defined as “a medical intervention involving the alteration of genetic material within living cells”, does not categorize mRNA vaccines [75]. We anticipate that the increasing number of product submissions will lead to the creation of regulatory standards explicitly tailored for therapeutic RNA products. The US regulations diverge from those in Europe. The EU classifies all these items as biological pharmaceuticals, except for peptides consisting of fewer than 40 amino acids, which it classifies as chemicals and proteins.

Given that numerous chemical medications have biological properties, it is essential to critically examine whether we should categorize mRNA products as chemicals or biologicals. However, if the mRNA produces a protein, we should either categorize it or use its translational result to classify the mRNA.

### 7.1. Biosimilars and Generics

The era of biosimilars, as the patents began to expire for hundreds of protein genes, was expected to expand the availability of these proteins. Still, the FDA has only approved 16 molecules [76] as biosimilars out of 100+ available options due mainly to the high development costs of biosimilars [42,77], drawing the attention of developers to products with significant markets. The definition of biosimilars mandates that they should have the same routes of administration, chemical structure, dosing, and indications, disqualifying mRNA-delivered proteins as biosimilars.

There is also no example of an mRNA product approved to deliver therapeutic protein, so recently, at the request of the author, the FDA has proposed naming as follows: Full name of therapeutic protein, mRNA (xxx), injection. For example, mRNA used to deliver darbepoetin alfa will be named as follows: farbepoetin alfa, mRNA (XXXX), injection. Developing these products faces newer challenges in addition to the standard considerations of toxicology due to their LNP delivery; one such constraint is to calculate the dose of the mRNA to deliver an appropriate dose of the protein, either for therapeutic or vaccination purposes. The FDA recommends a Phase 0 study where the dosing will be calculated based on animal models, such as rodents, which are expected to express a higher number of protein molecules for each molecule of mRNA injected. Clinical efficacy testing can be negotiated with the FDA if the mRNA product replicates the pharmacokinetic profile.

Consequently, an mRNA delivery system is considered a new biological drug filed under 351(a) application. However, the FDA will treat this as a “hybrid biosimilar”, as the toxicology of the active ingredient may not be required, and the nonclinical toxicology will mainly focus on the toxicity of the LNP formulation.

### 7.2. Copy of Licensed mRNA Product

While the FDA has only approved COVID-19 mRNA vaccines, it is anticipated that many mRNA products, both therapeutic and vaccines, will soon be approved [78]:

- Infectious diseases: Moderna is advancing mRNA vaccines for influenza (mRNA-1010), respiratory syncytial virus (RSV) with mRNA-1345, and cytomegalovirus (CMV) with mRNA-1647, all of which are in phase 2 or 3 trials. Other early-phase products target Zika, herpes, Lyme disease, and Nipah virus.
- Cancer: There is significant work in cancer vaccines, such as Moderna’s individualized neoantigen therapies (e.g., mRNA-4157), which target melanoma, non-small cell lung cancer, and renal cell carcinoma. These therapies are based on patient-specific tumor antigens and are in late-stage clinical development.
- Rare diseases: mRNA therapeutics are also being explored for metabolic and rare genetic diseases, including treatments for propionic acidemia (mRNA-3927) and methylmalonic acidemia (mRNA-3705). These therapies aim to replace or enhance deficient enzymes; a novel application for mRNA beyond traditional vaccines.
- Combination vaccines: Moderna and Pfizer are developing combination mRNA vaccines to simultaneously address multiple viruses, such as the flu and COVID-19 vaccine (mRNA-1083), which are progressing through phase 3 trials.

Once an mRNA product is licensed (within the gene therapy category as a biological drug), developers will have a unique opportunity to determine their sequence of nucleosides and copy them to provide an “identical” product. Ideally, this should be considered as an NDA product for chemical generics, since there is no difference in the chemistry with the reference product. The FDA has yet to face such a challenge, so a copy of an mRNA product will be treated as a new biological drug. However, exploiting the FDA’s recent GASK (Generally Accepted Scientific Knowledge) guideline, a developer will be able to challenge the regulatory pathway for these products [79]. However, these considerations will be met with strong opposition from big pharma for apparent reasons.

## 8. Conclusions

The thousands of proteins that control the delicate functions of the human body are yet to be identified, and their deficiencies addressed with therapies. The same broad focus is developing novel vaccines, including multi-target vaccination possibilities.

While the cost of establishing a manufacturing facility that is GMP compliant will always be high, the cost of establishing an mRNA facility should always be lower due to the simplicity of the manufacturing process. Over time, as PCR technology becomes available on a larger scale, the entire process of mRNA product manufacturing will be chemical. The cost of goods produced via mRNA technology will always be a small fraction of the recombinant protein cost, and the time to market will be months rather than years.

mRNA technology offers a rare opportunity for developing nations to secure their supply of therapeutic proteins and protein vaccines that will be subject to more minor safety concerns than if these products were manufactured via recombinant processes. Currently, 80% of the world cannot afford these therapeutic proteins and protein vaccines. However, we can fill this gap with confidence in the product’s safety, bringing remarkable benefits to humanity.

## Figures and Tables

**Table 1:** FDA-approved RPs [33].

Therapeutic RP	Brand Example	Application	Mechanism/Function
Adalimumab	Humira	Rheumatoid arthritis, psoriasis	TNF-alpha inhibitor
Aflibercept	Eylea	Age-related macular degeneration	VEGF inhibitor
Albutrepenonacog Alfa	Idelvion	Hemophilia B	Recombinant factor IX
Aldesleukin	Proleukin	Metastatic renal cell carcinoma	Interleukin-2 analog
Alglucosidase Alfa	Myozyme, Lumizyme	Pompe disease	Enzyme replacement therapy
Alirocumab	Praluent	Hypercholesterolemia	PCSK9 inhibitor
Anakinra	Kineret	Rheumatoid arthritis	IL-1 receptor antagonist
Asparaginase	Elspar	Acute lymphoblastic leukemia	An enzyme that depletes asparagine
Atezolizumab	Tecentriq	Various cancers	PD-L1 inhibitor
Avalglucosidase Alfa	Nexviazyme	Pompe disease	Enzyme replacement therapy
Avatrombopag	Doptelet	Thrombocytopenia	Thrombopoietin receptor agonist
Belatacept	Nulojix	Organ transplant rejection	Selective T cell co-stimulation blocker
Belimumab	Benlysta	Systemic lupus erythematosus	BAFF inhibitor
Bevacizumab	Avastin	Various cancers (e.g., colorectal, lung)	VEGF inhibitor
Blinatumomab	Blincyto	Acute lymphoblastic leukemia	BiTE targeting CD19 and CD3
Blisibimod	A-623	Systemic lupus erythematosus	BAFF inhibitor
Bremelanotide	Vyleesi	Hypoactive sexual desire disorder	Melanocortin receptor agonist
Brodalumab	Siliq	Psoriasis	IL-17 receptor antagonist
Burosumab	Crysvita	X-linked hypophosphatemia	FGF23 inhibitor
Canakinumab	Ilaris	Periodic fever syndromes	IL-1 beta inhibitor
Caplacizumab	Cablivi	Thrombotic thrombocytopenic purpura	von Willebrand factor inhibitor
Cenegermin	Oxervate	Neurotrophic keratitis	Recombinant human nerve growth factor
Crizanlizumab	Adakveo	Sickle cell disease	P-selectin inhibitor
Darbepoetin Alfa	Aranesp	Anemia (chronic kidney disease)	Stimulates red blood cell production
Denosumab	Prolia, Xgeva	Osteoporosis, bone cancer	RANKL inhibitor
Dornase Alfa	Pulmozyme	Cystic fibrosis	Mucolytic agent
Dostarlimab	Jemperli	Mismatch repair-deficient cancers	PD-1 inhibitor
Dupilumab	Dupixent	Atopic dermatitis, asthma	IL-4 receptor alpha antagonist
Durvalumab	Imfinzi	Lung cancer	PD-L1 inhibitor
Eculizumab	Soliris	Paroxysmal nocturnal hemoglobinuria	Complement inhibitor
Efgartigimod	Vyvgart	Myasthenia gravis	FcRn antagonist
Eltrombopag	Promacta	Thrombocytopenia	Thrombopoietin receptor agonist
Emicizumab	Hemlibra	Hemophilia A	Bispecific factor IXa- and X-directed antibody
Erenumab	Aimovig	Migraine prevention	CGRP receptor antagonist
Erythropoietin	Epogen, Procrit	Anemia (chronic kidney disease)	Stimulates red blood cell production
Etanercept	Enbrel	Autoimmune diseases	TNF receptor fusion protein
Etrolizumab	In development	Ulcerative colitis	Anti-beta7 integrin antibody
Evolocumab	Repatha	Hypercholesterolemia	PCSK9 inhibitor
Factor IX	BeneFIX, Idelvion	Hemophilia B	Clotting factor replacement
Factor VIII	Advate, Eloctate	Hemophilia A	Clotting factor replacement
Filgrastim	Neupogen	Neutropenia	Stimulates white blood cell growth
Fremanezumab	Ajovy	Migraine prevention	CGRP inhibitor
Galcanezumab	Emgality	Migraine prevention	CGRP inhibitor
Givosiran	Givlaari	Acute hepatic porphyria	RNA interference (RNAi) agent targeting ALAS1
Golimumab	Simponi	Rheumatoid arthritis, psoriatic arthritis	TNF-alpha inhibitor
Ibalizumab-uiyk	Trogarzo	HIV-1 infection	CD4-directed post-attachment inhibitor
Idursulfase	Elaprase	Hunter syndrome	Enzyme replacement therapy
Imiglucerase	Cerezyme	Gaucher disease	Enzyme replacement therapy
Imlifidase	Idefirix	Desensitization in kidney transplantation	IgG-degrading enzyme
Inclisiran	Leqvio	Hypercholesterolemia	siRNA targeting PCSK9
Infliximab	Remicade	Crohn’s disease, rheumatoid arthritis	TNF-alpha inhibitor
Insulin	Humulin, Novolin	Diabetes	Blood sugar regulation
Interferon Beta-1a	Avonex, Rebif	Multiple sclerosis	Immune modulation
Ipilimumab	Yervoy	Melanoma	CTLA-4 inhibitor
Lanadelumab	Takhzyro	Hereditary angioedema	Kallikrein inhibitor
Lanreotide	Somatuline Depot	Acromegaly	Somatostatin analog
Laronidase	Aldurazyme	Mucopolysaccharidosis I	Enzyme replacement therapy
Leronlimab	In development	HIV, cancer	CCR5 antagonist
Luspatercept	Reblozyl	Anemia in beta-thalassemia	Erythroid maturation agent
Maribavir	Livtencity	CMV infection post-transplant	UL97 protein kinase inhibitor
Natalizumab	Tysabri	Multiple sclerosis, Crohn’s disease	Integrin inhibitor
Niraparib	Zejula	Ovarian cancer	PARP inhibitor
Nivolumab	Opdivo	Melanoma, lung cancer	PD-1 inhibitor
Ocrelizumab	Ocrevus	Multiple sclerosis	Anti-CD20 monoclonal antibody
Olaparib	Lynparza	BRCA-mutated cancers	PARP inhibitor
Omalizumab	Xolair	Asthma	IgE inhibitor
Palivizumab	Synagis	Respiratory syncytial virus (RSV)	RSV-specific monoclonal antibody
Pegloticase	Krystexxa	Chronic gout	Uric acid breakdown enzyme
Pegzilarginase	AEB1102	Arginase deficiency	Recombinant human arginase
Pembrolizumab	Keytruda	Melanoma, lung cancer	PD-1 inhibitor
Plerixafor	Mozobil	Stem cell mobilization	CXCR4 antagonist
Ranibizumab	Lucentis	Diabetic macular edema	VEGF inhibitor
Raxibacumab	Abthrax	Anthrax infection	Anthrax toxin inhibitor
Rilonacept	Arcalyst	Cryopyrin-associated periodic syndromes	IL-1 inhibitor
Risankizumab	Skyrizi	Psoriasis	IL-23 inhibitor
Risdiplam	Evrysdi	Spinal muscular atrophy	SMN2 splicing modifier
Rituximab	Rituxan	Non-Hodgkin’s lymphoma	CD20-targeted antibody
Romiplostim	Nplate	Thrombocytopenia	Thrombopoietin receptor agonist
Romosozumab	Evenity	Osteoporosis	Sclerostin inhibitor
Satralizumab	Enspryng	Neuromyelitis optica	IL-6 receptor antagonist
Secukinumab	Cosentyx	Psoriasis, ankylosing spondylitis	IL-17A inhibitor
Sutimlimab	Enjaymo	Cold agglutinin disease	Complement C1s inhibitor
Teplizumab	Tzield	Type 1 diabetes (delay onset)	Anti-CD3 antibody
Teprotumumab	Tepezza	Thyroid eye disease	IGF-1 receptor antagonist
Tildrakizumab	Ilumya	Psoriasis	IL-23 inhibitor
Tocilizumab	Actemra	Rheumatoid arthritis	IL-6 receptor antagonist
Tralokinumab	Adtralza	Atopic dermatitis	IL-13 inhibitor
Trastuzumab	Herceptin	HER2-positive breast cancer	Targets HER2 receptor
Ustekinumab	Stelara	Psoriasis, Crohn’s disease	IL-12 and IL-23 inhibitor
Vedolizumab	Entyvio	Ulcerative colitis, Crohn’s disease	Integrin antagonist
Vibecotolimab	In development	Cancer immunotherapy	TIM-3 inhibitor
Vilobelimab	Vilova	Sepsis	C5a inhibitor

**Table 2:** FDA-licensed RP vaccines [53].

Vaccine	Brand Example	Target Protein	Prevention of
Hepatitis B	Engerix-B, Recombivax HB	Hepatitis B surface antigen (HBsAg)	Hepatitis B virus infection
Human Papillomavirus (HPV)	Gardasil, Cervarix	L1 protein from various HPV types (e.g., 6, 11, 16, and 18)	HPV-related cancers and genital warts
Influenza (Flu)	Flublok Quadrivalent	Hemagglutinin protein from multiple influenza strains	Seasonal influenza
SARS-CoV-2 (COVID-19)	NVX-CoV2373 (Novavax)	Spike (S) protein of SARS-CoV-2	COVID-19
Meningococcal B	Trumenba, Bexsero	Factor H binding protein (fHbp), NHBA, and others	Neisseria meningitidis serogroup B infection
Malaria	Mosquirix (RTS, S)	Circumsporozoite protein (CSP) of Plasmodium falciparum	Malaria in children
Lyme Disease	LYMErix (discontinued)	Outer surface protein A (OspA) of Borrelia burgdorferi	Lyme disease (was discontinued)
Rabies	Rabivax-S	Glycoprotein G of rabies virus	Rabies, typically for post-exposure prophylaxis
Ebola Virus	Ervebo (primarily viral vector)	Glycoprotein from the Ebola virus	Ebola virus disease
Herpes Zoster (Shingles)	Shingrix	Glycoprotein E of the varicella-zoster virus	Shingles (herpes zoster) in older adults
Dengue Virus	Dengvaxia	Envelope and membrane proteins from multiple dengue virus serotypes	Dengue virus in endemic areas
Pertussis Component of DTaP	Infanrix, Daptacel	Pertussis toxin, filamentous hemagglutinin, pertactin (in acellular vaccines)	Pertussis (whooping cough), especially in children
RSV (Respiratory Syncytial Virus)	In development	F protein (fusion protein) of RSV	RSV infection, especially in infants and the elderly
Yellow Fever	In development	Envelope proteins of the yellow fever virus	Yellow fever
HIV	In development	Envelope glycoprotein (gp120) or gp160 of HIV	HIV infection
Leishmaniasis	In development	Kinetoplastid membrane protein (KMP-11) and others for Leishmania species	Leishmaniasis, particularly in endemic areas
Chikungunya Virus	In development	Envelope protein of chikungunya virus	Chikungunya virus infection

**Table 3:** Comparative analysis of RPs vs. IMPs.

Step	Recombinant Protein Engineering	mRNA Technology	Comparison
1. Research and development	High: Extensive work on gene design, host cell selection, and vector optimization.	Moderate: mRNA sequence design and modification (e.g., codon optimization, UTR engineering).	Recombinant costs higher due to host cell studies.
2. Plasmid construction	High: Stable plasmid generation for host cells (e.g., CHO cells).	Moderate: Plasmid or template synthesis for IVT (in vitro transcription).	Recombinant cost higher for stable plasmids.
3. Cell line development	Very high: Creation of stable expression systems (e.g., CHO, *E. coli*).	None: mRNA avoids the need for cell lines.	Recombinant is far more expensive.
4. Upstream production	High: Large-scale bioreactors for fermentation and cell culture.	Low: Scalable IVT reactions in cell-free systems.	Recombinant is costlier due to cell culture.
5. Downstream purification	Very high: Protein purification steps like chromatography, filtration, and refolding.	Moderate: RNA purification (e.g., chromatography, ultrafiltration).	Recombinant is more intensive and costly.
6. Formulation development	Moderate: Stabilizing proteins (e.g., lyophilization, additives).	Moderate: Stabilization of mRNA (e.g., lipid nanoparticle [LNP] formulation).	Similar costs.
7. Scalability	High: Requires optimized bioreactor and downstream processes.	Low: IVT is inherently scalable with fewer bottlenecks.	mRNA is more scalable and cost-effective.
8. Quality control and testing	Very high: Protein structure/function analysis, glycosylation profiling, etc.	Moderate: Sequencing, purity checks, and potency assays for mRNA.	Recombinant involves complex QC steps.
9. Regulatory approval costs	High: Extensive CMC data and biosimilar comparability studies.	High: Emerging guidelines but fewer comparability studies are needed.	Comparable costs.
10. Manufacturing cost per dose	Very high: Dependent on yields, process efficiency, and scalability.	Low: Cost-efficient once IVT and LNP formulation are optimized.	mRNA is cheaper per dose.
11. Time to market	Long: Several years (5–10 years) due to cell line development and process optimization.	Short: 1–3 years due to faster development and more straightforward production.	mRNA faster to market.
Total cost estimate	High cost per unit, especially for low-yield, complex proteins (can exceed hundreds of millions in total development costs for a commercial product).	Lower overall costs due to reduced infrastructure needs and simpler production processes (~30–50% less expensive than recombinant protein production).	An affordable option for the majority of the world.

**Table 4:** mRNA developers.

Company Name	Headquarters	Key mRNA Products/Research Areas	Notable Collaborations
Arcturus Therapeutics	San Diego, CA, USA	mRNA-based vaccines and therapeutics, including COVID-19 vaccine candidates	Duke–NUS Medical School (COVID-19 vaccine development)
BioNTech	Mainz, Germany	COVID-19 vaccine (Comirnaty), cancer immunotherapies, infectious disease vaccines	Pfizer (COVID-19 vaccine development)
Chimeron Bio	Philadelphia, PA, USA	mRNA-based vaccines and therapeutics using the proprietary ChaESAR platform	None specified
CureVac	Tübingen, Germany	mRNA-based vaccines and therapeutics for infectious diseases and cancer	GSK (influenza and COVID-19 vaccines)
eTheRNA Immunotherapies	Niel, Belgium	mRNA-based immunotherapies for cancer and infectious diseases	None specified
Ethris	Planegg, Germany	mRNA-based therapeutics for respiratory diseases	None specified
GSK (GlaxoSmithKline)	Brentford, UK	mRNA vaccines for influenza and COVID-19	CureVac (mRNA vaccine development)
Laronde	Cambridge, MA, USA	Endless RNA™ (eRNA) therapeutics for various diseases	None specified
Moderna	Cambridge, MA, USA	COVID-19 vaccine (Spikevax), RSV vaccine (Mresvia), cancer vaccines, rare disease therapeutics	None specified
Nutcracker Therapeutics	Emeryville, CA, USA	mRNA-based cancer treatments	None specified
Omega Therapeutics	Cambridge, MA, USA	mRNA-based epigenomic programming for various diseases	None specified
Pfizer	New York, NY, USA	mRNA-based COVID-19 vaccine (Comirnaty)	BioNTech (COVID-19 vaccine development)
RaNA Therapeutics	Cambridge, MA, USA	mRNA-targeted therapies for genetic diseases	None specified
Replicate Bioscience	San Diego, CA, USA	Self-replicating RNA therapeutics for cancer and autoimmune diseases	None specified
RNAimmune	Gaithersburg, MD, USA	mRNA-based therapies for cancer, rare diseases, and prophylactic vaccines	None specified
Sanofi	Paris, France	mRNA vaccines and therapeutics for infectious diseases	Acquired Translate Bio
Strand Therapeutics	Cambridge, MA, USA	mRNA-based therapeutics with programmable control for cancer and other diseases	None specified
Tiba Biotech	Cambridge, MA, USA	mRNA vaccines and therapeutics using novel nanoparticle delivery systems	None specified
Translate Bio	Lexington, MA, USA	mRNA therapeutics for various diseases, including cystic fibrosis and infectious diseases	Sanofi (acquired Translate Bio)
Vaxart	South San Francisco, CA, USA	Oral recombinant vaccines, including mRNA-based candidates for COVID-19 and other viruses	None specified
